# Ceramide phosphoethanolamine synthase SMSr is a target of caspase-6 during apoptotic cell death

**DOI:** 10.1042/BSR20170867

**Published:** 2017-07-17

**Authors:** Birol Cabukusta, Niclas T. Nettebrock, Matthijs Kol, Angelika Hilderink, Fikadu G. Tafesse, Joost C.M. Holthuis

**Affiliations:** 1 Molecular Cell Biology Division, Faculty of Biology/Chemistry, University of Osnabrück, 49076 Osnabrück, Germany; 2 Oregon Health and Science University, Portland, OR 97239, U.S.A.; 3 Membrane Biochemistry and Biophysics, Bijvoet Center and Institute of Biomembranes, Utrecht University, 3584 CH Utrecht, the Netherlands

**Keywords:** apoptosis, caspases, membrane biochemistry, sphingolipids

## Abstract

Ceramides are essential precursors of sphingolipids with a dual role as mediators of apoptotic cell death. Previous work revealed that the ER-resident ceramide phosphoethanolamine (CPE) synthase SMSr/SAMD8 is a suppressor of ceramide-mediated apoptosis in cultured cells. Anti-apoptotic activity of SMSr requires a catalytically active enzyme but also relies on the enzyme’s N-terminal sterile α-motif or SAM domain. Here, we demonstrate that SMSr itself is a target of the apoptotic machinery. Treatment of cells with staurosporine or the death receptor ligand FasL triggers caspase-mediated cleavage of SMSr at a conserved aspartate located downstream of the enzyme’s SAM domain and upstream of its first membrane span. Taking advantage of reconstitution experiments with SMSr produced in a cell-free expression system, specific caspase-inhibitors and gene silencing approaches, we show that SMSr is a novel and specific substrate of caspase-6, a non-conventional effector caspase implicated in Huntington’s and Alzheimer’s diseases. Our findings underscore a role of SMSr as negative regulator of ceramide-induced cell death and, in view of a prominent expression of the enzyme in brain, raise questions regarding its potential involvement in neurodegenerative disorders.

## Introduction

Apoptosis is a form of programmed cell death fundamental to organismal development and tissue homeostasis. A defective apoptotic machinery contributes to tumor progression, autoimmune diseases, and viral infections, while excessive apoptosis can cause neurodegenerative disorders [1-4]. Execution of apoptosis is orchestrated by caspases, a specific class of proteolytic enzymes that are synthesized as inactive zymogens and then activated by either autocatalytic cleavage or cleavage by other caspases [5]. Caspase-mediated cleavage of specific target proteins results in either activation of proteins that directly participate in the execution phase of apoptotic cell death or inhibition of proteins that normally promote cell survival. Caspases are categorized as initiator, executioner, or inflammatory caspases, according to their distinct roles. Apoptosis can be initiated through extrinsic or intrinsic signaling pathways. Activation of the extrinsic pathway involves stimulation of death receptors on the cell surface by ligands such as FasL. This results in recruitment, dimerization, and self-activation of initiator caspase-8 and -10, which then propagate the apoptosis signal by cleaving the downstream executioner caspase-3 and -7 [6]. The intrinsic pathway can be activated by various stress stimuli (e.g. DNA damage, cytokines) and is initiated by permeabilization of the outer mitochondrial membrane to intermembrane space proteins like cytochrome *c.* The release of cytochrome *c* into the cytosol leads to formation of the apoptosome and subsequent recruitment, dimerization, and self-activation of initiator caspase-9, which then cleaves and activates caspase-3 and -7 [6,7]. Caspase-6 is activated by caspase-3 and can cleave caspase-8. Moreover, caspase-6 is capable of self-cleavage and activation, suggesting that the enzyme can assume simultaneous roles as executioner and initiator caspase [6].

A growing body of evidence indicates that ceramides, central intermediates of sphingolipid metabolism, act as potent mediators of apoptotic cell death [8,9]. Ceramides can be generated *de novo* by ceramide synthases in the ER [10,11] or through breakdown of sphingomyelin (SM) by sphingomyelinases that operate in the cytosol, in lysosomes, or on the cell surface [9]. Numerous studies have demonstrated that cellular ceramide levels rise in response to various apoptotic stimuli including staurosporine [12], tumor necrosis factor (TNF)α [13], death receptor ligand FasL [14,15], and irradiation [16] through activation of sphingomyelinases, stimulation of *de novo* ceramide synthesis, or both. Interventions that suppress ceramide accumulation render cells resistant to these apoptotic stimuli, indicating that ceramides are necessary and sufficient to trigger apoptosis [17-22]. Consequently, targeting the enzymes involved in ceramide metabolism has emerged as a new approach in anti-cancer therapy [23,24].

Not only the abundance of ceramides *per se*, but also their subcellular distribution has a major impact on cellular life–death decision making. A deregulation of ceramide levels in the ER has frequently been linked to induction of mitochondrial apoptosis. While some studies indicate that ceramides influence this process by sensitizing cells to ER stress and activating apoptotic regulators of the unfolded protein response, experiments with isolated mitochondria suggest that newly synthesized ceramides are able to initiate the execution phase of apoptosis by directly promoting mitochondrial outer membrane permeabilization [25]. Indeed, recent work demonstrated that targeting newly synthesized ER ceramides to mitochondria suffices to trigger apoptotic cell death [26]. Although some studies provide evidence that ceramides can form pores in the outer membrane of isolated mitochondria that are large enough to allow passage of cytochrome *c* [27,28], the mechanism by which ceramides trigger mitochondrial apoptosis remains to be established.

The bulk of newly synthesized ceramides in mammalian cells is converted into SM by an SM synthase (SMS) in the lumen of the *trans*-Golgi [29]. Efficient production of SM requires the cytosolic ceramide transfer protein (CERT), which mediates non-vesicular delivery of ER ceramides to the *trans*-Golgi [30]. Identification of the Golgi-resident SM synthase uncovered a multiplicity of SMS-encoding genes in the human genome [29,31]. Mammalian cells contain two SMS isoforms, namely SMS1 in the *trans*-Golgi and SMS2 at the plasma membrane [31,32]. Together with SMS-related protein SMSr (also known as SAMD8), they constitute the SMS enzyme family [29]. SM synthases catalyze the interconversion of phosphatidylcholine and ceramide with SM and diacylglycerol. Consequently, these enzymes occupy a central position at the crossroads of sphingolipid and glycerophospholipid metabolism and have considerable biological potential as regulators of the balance between the pro-apoptotic lipid ceramide and the pro-survival lipid diacylglycerol. In line with this concept, overexpression of SMS1 protects cells from photodynamic therapy and FasL-induced apoptotic cell death, whereas its depletion sensitizes cells for the same treatments [33-35]. Moreover, SMS1 has been identified as a target of caspases in FasL-treated leukemia cells. Caspase-mediated cleavage of SMS1 occurs downstream of caspase-8 and results in the inhibition of SM biosynthesis. The subsequent accumulation of ceramides is believed to enhance FasL-induced activation of caspase-9 and apoptotic cell death [35]. Thus, SMS1 appears an attractive therapeutic target to sensitize diseased cells for the induction of apoptosis.

SMSr is by far the best conserved member of the SMS family, with structural homologs found in organisms that do not synthesize SM like *Drosophila* [36,37]. Indeed, SMSr is not a conventional SM synthase but instead produces trace amounts of the SM analog ceramide phosphoethanolamine (CPE) in the lumen of the ER [36]. The enzyme is ubiquitously expressed in mammalian tissues, with a strong expression in brain, testis, kidney, and pancreas [38]. We previously reported that acute disruption of SMSr catalytic activity in cultured mammalian cells causes a substantial rise in ER ceramides and their mislocalization to mitochondria, triggering mitochondrial apoptosis [36,39]. In addition, we found that SMSr-catalyzed CPE production, although required, is not sufficient to suppress ceramide-induced cell death and that SMSr-mediated ceramide homeostasis is critically dependent on the enzyme’s N-terminal sterile α motif or SAM domain. Based on these results, we postulated that SMSr serves a role in monitoring ER ceramide levels to prevent untimely cell death during sphingolipid biosynthesis [39]. Considering its anti-apoptotic activity, SMSr would qualify as a rational target of the apoptotic machinery, analogous to SMS1. In the present study, we experimentally verified this prediction.

## Experimental

### Chemicals and antibodies

Staurosporine and cyclohexamide were from Sigma–Aldrich, z-VAD-fmk from Calbiochem, z-VEID-fmk and SuperFasLigand-FLAG from Enzo, Ni^2+^-NTA agarose from QIAGEN, goat polyclonal anti-V5 agarose from Bethyl, active recombinant human caspases from BioVision, and WEPRO2240 wheat germ extract from Cell-free Sciences. Wheat germ phosphatidylinositol was from Lipid Products U.K. and egg phosphatidylcholine and synthetic dioleoylphosphatidylethanolamine were from Avanti Polar Lipids. The following antibodies were used: mouse monoclonal anti-V5 (R960-25, 1:4000; Invitrogen), mouse monoclonal anti-PARP1 (sc8007, 1:1000; Santa Cruz), rabbit polyclonal anti-caspase-9 (6502S, 1:700, Cell Signaling), rabbit polyclonal anti-caspase-3 (A303-657A-T, 1:1000; Bethyl), rabbit polyclonal anti-caspase-6 (9762, 1:1000, Cell Signaling), mouse monoclonal anti-actin (A1978, 1:10,000; Sigma–Aldrich), sheep polyclonal anti-TGN46 (AHP500, 1:200, AbD Serotec), rabbit polyclonal anti-calnexin (sc11397, 1:1000; Santa Cruz), mouse monoclonal anti-ERGIC-53 (NBP2-03381, 1:500, Novus bio), rabbit polyclonal anti-lamin A/C (1:1000, sc-20681, Santa Cruz), goat polyclonal anti-rabbit HRP (1:4000, 31460, Thermo), goat polyclonal anti-mouse HRP (1:4000, 31430, Thermo), donkey polyclonal anti-mouse Cy3 (715-165-150, 1:400, Jackson ImmunoResearch), donkey polyclonal anti-rabbit Cy5 (711-175-152, 1:400, Jackson ImmunoResearch), and donkey polyclonal anti-Sheep/Goat FITC (STAR88F, 1:200, AbD Serotec).

### DNA constructs

For mammalian expression of C-terminal V5/His6-tagged human SMSr, the corresponding cDNA was PCR amplified and cloned into pcDNA3.1/V5-His TOPO (Invitrogen) according to the manufacturer’s instructions. For cell-free expression, the ORF of SMSr was PCR-amplified in-frame with a C-terminal V5 epitope and cloned into the wheat germ pEU-Flexi expression vector (kind gift of Brian G. Fox and James D. Bangs, University of Wisconsin, Madison) [40]. For the establishment of HeLa cell lines stably transduced with SMSr expression constructs, the ORF of SMSr with a C-terminal V5 epitope was cloned into retroviral expression vector pLNCX2 (Clonetech). Single amino acid substitutions were introduced by site-directed mutagenesis using the megaprimer PCR method [41].

### Cell culture and transfection

HeLa cells (ATCC-CCL2) were cultured in high glucose DMEM supplemented 10% FBS. Cells were transfected with DNA constructs using Effectene (Qiagen) according to instructions of the manufacturer. Twenty-four hours post-transfection, the cells were treated with 1 μg/ml staurosporine for 6 h or treated with 100 ng/μl FasL and 18.75 μM cyclohexamide for 4 h in the presence or absence of 20 μM z-VAD-fmk. Next, floating and adherent cells were collected and lysed in lysis buffer (1% TritonX-100, 1 mM EDTA (pH 8.0), 150 mM NaCl, 20 mM Tris (pH 7.5), and 10 mM *N*-ethyl maleimide) supplemented with protease inhibitor cocktail (1 μg/ml aprotinin, 1 μg/ml leupeptin, 1 μg/ml pepstatin, 5 μg/ml antipain, and 157 μg/ml benzamidine). Nuclei were removed by centrifugation at 15000 ***g*** for 10 min at 4°C. Post-nuclear supernatants were incubated with pre-washed anti-V5 agarose beads or Ni^2+^-NTA agarose beads for 3 h at 4°C. Beads were washed three times with lysis buffer and eluted in SDS sample buffer (62.5 mM Tris (pH 6.8), 2% SDS, 10% glycerol, 0.005% Bromophenol Blue) at 95°C for 5 min. Eluates were subjected to immunoblotting.

### Generation of cell lines stably expressing SMSr-V5 and SMSr^CR^-V5

HeLa cells stably expressing V5-tagged SMSr and SMSr^CR^ were created by retroviral transduction. To this end, low-passage human HEK293T cells (ATCC ® CRL-3216™) grown in DMEM supplemented with 10% serum were co-transfected with SMSr-V5/pLNCX2 or SMSr^CR^-V5/pLNCX2 and packaging vectors using Lipofectamine 2000 (Thermo Fisher). After 48 h, supernatants were harvested, passed through a 0.45 μm filter, and the virus-containing medium was used to transduce HeLa cells. The cells were cultured in DMEM supplemented with 10% serum and 0.8 mg/μl geneticin (G418). Expression of V5-tagged proteins was confirmed by indirect immunofluorescence and Western blotting using V5 antibody. For *in vivo* inhibition of CASP6-mediated SMSr cleavage, cells were treated with 1.5 μg/ml staurosporine for 6 h in the presence of 20 μM z-VAD-fmk, 4 μM z-VEID-fmk, or DMSO for 6 h. For the time course experiments of staurosporine- and FasL-induced apoptosis, cells were treated with 1μg/ml staurosporine or 50 ng/μl FasL and 18.75 μM cyclohexamide for indicated times. Floating and adherent cells were collected and lysed in lysis buffer. Nuclei were removed by centrifugation at 15000 ***g*** for 10 min at 4°C. Post-nuclear supernatants were subjected to immunoblotting.

### siRNA transfection and immunoblotting

RNA interference was performed on HeLa cells stably expressing V5-tagged SMSr using Oligofectamine (Invitrogen) in Opti-MEM I Glutamax medium (Invitrogen) as described previously [39]. Hs_CASP6_8 (SI02662450) and Hs_CASP3_7 (SI02654603) siRNAs were from QIAGEN. Nonsense (NS) siRNA (QIAGEN) target sequence was: 5′-AAUUCUCCGAACGUGUCACGU-3′. Seventy-two hours after the start of siRNA treatment, cells were treated with staurosporine (1.5 μg/ml) for an additional 6 h. Next, cells were lysed in lysis buffer and nuclei were separated by centrifugation at 15000 ***g*** for 10 min at 4°C. Post-nuclear supernatant samples were mixed with 2× SDS sample buffer (125mM Tris (pH 6.8), 4% SDS, 20% glycerol, 0.01% Bromophenol Blue) and incubated at 95°C for 5 min. Samples were subjected to SDS/PAGE and proteins were transferred on PVDF membranes. Membranes were incubated with blocking, primary antibody and HRP-conjugated secondary antibody solutions respectively. Images were acquired with BIO-RAD ChemiDoc XRS+ system and quantified using Image Lab 5.0 (BIO-RAD) software. Statistical analysis was performed using MATLAB (MathWorks) software.

### Cell-free production and caspase-mediated cleavage of SMSr

Cell-free production of SMSr-V5 in proteoliposomes was described previously [40]. Briefly, wheat germ expression vector SMSr-V5/pEU-Flexi was treated with Proteinase K to remove trace amounts of RNAse, purified by phenol/chloroform extraction, and dissolved at 1 μg/μl in water. *In vitro* transcription was carried out in 50 μl reaction volume containing 5 μg of plasmid DNA, 2 mM each of ATP, GTP, CTP, and UTP, 20 units of Sp6 RNA polymerase and 40 units of RNasin in 100 mM HEPES (pH 7.8), 25 mM Mg-acetate, 2 mM sperimidine, and 10 mM DTT. After incubation at 37°C for 4 h, the reaction mixture was centrifuged at 3400 ***g*** for 5 min at RT. The supernatant, containing SMS mRNA, was collected to set up a cell-free translation reaction. Twenty microliters of mRNA-containing mixture was used for 100 μl translation mixture containing 0.3 mM each of all 20 amino acids, 2 mM liposomes, 40 μg/ml creatine kinase, 15 OD_600_ WEPRO2240 wheat germ extract, 15 mM HEPES (pH 7.8), 50 mM potassium acetate, 1.25 mM Mg-acetate, 0.2 mM spermidine, 2 mM DTT, 0.6 mM ATP, 125 μM GTP, 8 mM creatine phosphate, and 0.0025% sodium azide. Unilamellar liposomes used in the translation reactions were prepared from a defined lipid mixture (PC/PE/PI, 2:2:1 mol%) in 25 mM HEPES (pH 7.5), 100 mM NaCl with a miniextruder (Avanti Polar Lipids). The reaction mixtures were incubated for 4 h at 26°C. Next, 2 μl of cell-free translation mixture was mixed with 10 μl of 2× caspase reaction buffer (1% CHAPS, 84 mM KCl, 10 mM MgCl_2_, 100 mM HEPES (pH 7.4), 100 mM NaCl, 20 mM EDTA, and 20 mM DTT), one to two units of recombinant human caspases in the presence of z-VAD-fmk (final concentration 1 mM), z-VEID-fmk (final concentration 100 μM), or DMSO in a total volume of 20 μl. Samples were incubated at 37°C for 2 h and then subjected to SDS/PAGE and immunoblot analysis using an anti-V5 antibody. To confirm the activity of recombinant human caspases, HeLa cells were lysed in 0.5% CHAPS, 42 mM KCl, 5 mM MgCl_2_, 50 mM HEPES (pH 7.4), 50 mM NaCl, and 10 mM EDTA. Nuclei were removed by centrifugation at 15000 ***g*** for 10 min at 4°C and DTT was added to a final concentration of 10 mM. Twenty microliters of post-nuclear supernatant (corresponding to ∼600,000 cells) was incubated with one unit active recombinant human caspase for 2 h at 37°C. Reactions were subjected to immunoblot analysis using anti-PARP1 and Lamin A/C antibodies.

### Immunofluorescence microscopy

HeLa cells stably transduced with V5-tagged SMSr expression constructs were seeded on glass coverslips. Cells were fixed using 4% (w/v) paraformaldehyde in PBS at RT and quenched using 25 mM NH_4_Cl in PBS. Cells were permeabilized using PBS containing 0.1% (w/v) saponin and 0.2% (w/v) BSA. Coverslips were immunostained using mouse anti-V5, sheep anti-TGN46, rabbit anti-calnexin primary antibodies followed by donkey anti-rabbit Cy5, donkey anti-mouse Cy3, and donkey anti-sheep/goat FITC antibodies. Coverslips were mounted with Prolong Gold Antifade Reagent (Thermo Fisher Scientific). Images were captured using a confocal microscope (Olympus LSM FV1000) equipped with two spectral and a single standard detector, an UPLSAPO 60x/NA 1.35 oil immersion objective (Olympus) and an Olympus laser box with AOTF laser combiner. Fluorophores were excited using 488, 559, and 635 nm lasers. Excitation light was reflected by a 405/488/559/635nm dichroic mirror. Emitted light was collected using secondary dichroic mirrors SDM-560 and SDM-640 and a barrier filter BA 655-755 for FITC, Cy3, and Cy5 respectively. To avoid cross-talk between image channels, images for different fluorophores were collected sequentially in a descending order of wavelength, i.e. longer to shorter wavelengths*.* Image analysis was performed using ImageJ (NIH) software.

## Results

### SMSr undergoes caspase-mediated cleavage at Asp120 in staurosporine-treated HeLa cells

**Figure 1:**
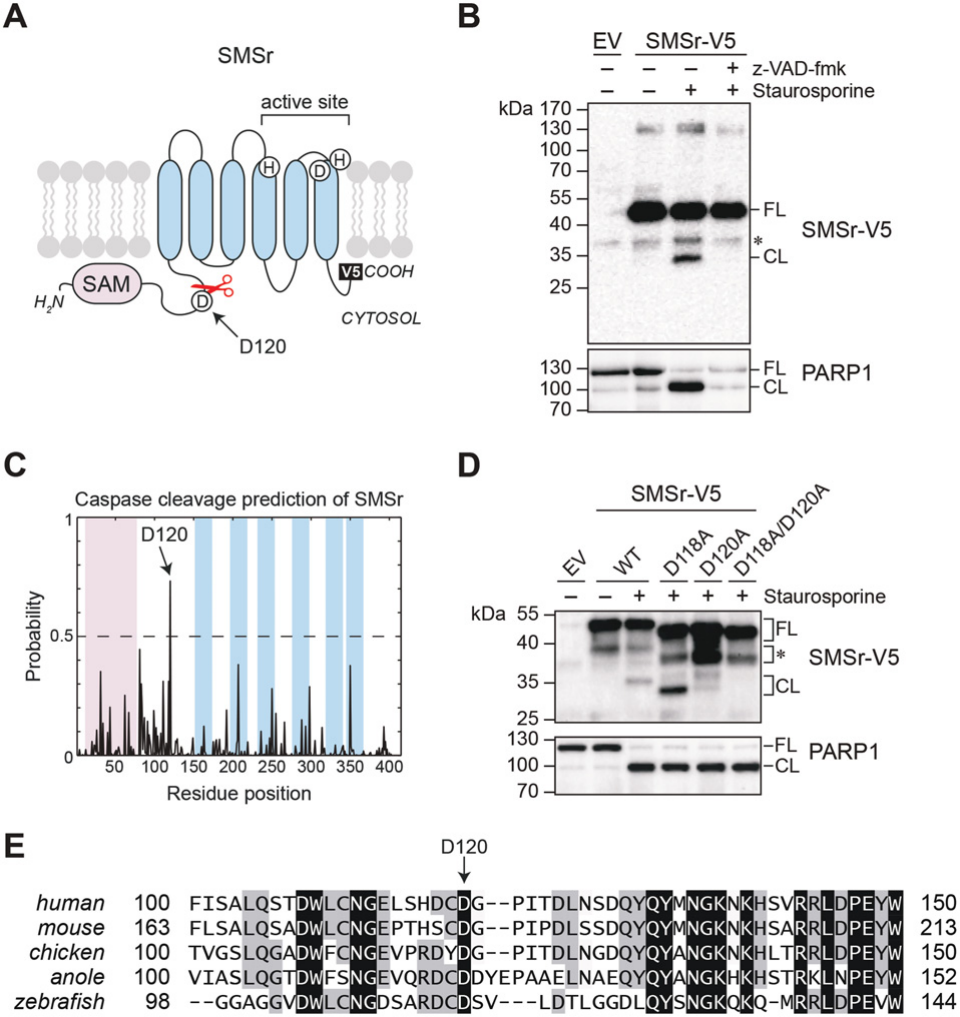
SMSr undergoes caspase-mediated cleavage at Asp120 in staurosporine-treated HeLa cells (**A**) Domain representation of the ER-resident membrane protein SMSr. Predicted membrane spans are marked in blue, whereas the N-terminal SAM domain is marked in pink. T. he positions of a predicted caspase cleavage site, Asp120, and C-terminal V5 epitope are also indicated. (**B**) HeLa cells transfected with empty vector (EV) or SMSr carrying a C-terminal V5/polyHis tag (SMSr-V5) were treated with 1 μg/ml staurosporine for 6 h in the presence or absence of 20 μM z-VAD-fmk, lysed, and subjected to immunoprecipitation using an anti-V5 antibody. Immunoprecipitates and total lysates were immunoblotted using an anti-V5 antibody (top) or anti-PARP1 antibody (bottom) respectively. A non-specific immunoreactive band is marked with an asterisk. FL, full length; CL, cleaved. (**C**) Cascleave, a caspase substrate cleavage site prediction tool [42], identifies Asp120 as the primary caspase cleavage site in human SMSr. Residue positions corresponding to the SAM domain are marked in pink and membrane spans in blue, as in (A). (**D**) HeLa cells transfected with V5/polyHis-tagged SMSr, SMSr^D118A^, SMSr^D120A^, or SMSr^D118A, D120A^ were treated with 1 μg/ml staurosporine for 6 h, lysed, and subjected to Ni^2+^-NTA chromatography. Eluates and total lysates were immunoblotted using an anti-V5 antibody (top) or anti-PARP1 antibody (bottom) respectively. An SMSr-V5-derived breakdown product formed independently of staurosporin-treatment is marked with an asterisk. (**E**) Sequence alignment of the region centered around the predicted caspase cleavage site in SMSr orthologs from various vertebrate species.

To determine whether SMSr is a target of the apoptotic machinery, HeLa cells expressing human SMSr with a C-terminal polyHis and V5 epitope were treated with staurosporine, lysed and then subjected to immunoblot analysis using anti-V5 antibody. Stauroporine-induced proteolytic cleavage of SMSr, yielding a V5-tagged fragment of ∼33 kDa (Figure 1A and B). Cleavage of SMSr was blocked by incubating cells with the pan-caspase inhibitor z-VAD-fmk, indicating that this process is dependent on caspases. Immunoblot analysis of staurosporine-treated cells expressing SMSr with an N-terminal V5 tag suggested that cleavage occurred at a single site downstream of the N-terminal SAM domain, but upstream of the first predicted membrane span (data not shown). This region contains two predicted caspase cleavage sites, namely Asp118 and Asp120 (Figure 1 C; [42]), with the latter showing the highest probability and strongest degree of conservation across distinct species (Figure 1E). To test which of these sites is cleaved in staurosporine-treated cells, each of the corresponding aspartates was substituted for an alanine. While substitution of Asp118 had no obvious impact, substitution of Asp120 virtually abolished staurosporine-induced cleavage of SMSr (Figure 1D). We noticed that substitution of Asp118 affected the apparent gel mobility of the V5-tagged cleavage fragment, which is remarkable as cleavage at Asp120 should give rise to the same C-terminal fragment in both SMSr-V5 and SMSr^D118A^-V5. Thus, it appears that substitution of Asp118 causes a shift in the capsase cleavage site. Moreover, we observed some residual cleavage of SMSr^D120A^-V5 in staurosporine-treated cells, which was eliminated when both Asp118 and Asp120 were substituted. The caspase-resistant form of SMSr, SMSr^D118A/D120A^-V5, is henceforth termed SMSr^CR^. From these data, we conclude that SMSr is a substrate of caspases that undergoes caspase-mediated cleavage primarily at Asp120 during staurosporine-induced apoptosis.

### Caspase-6 cleaves SMSr *in vitro*

**Figure 2:**
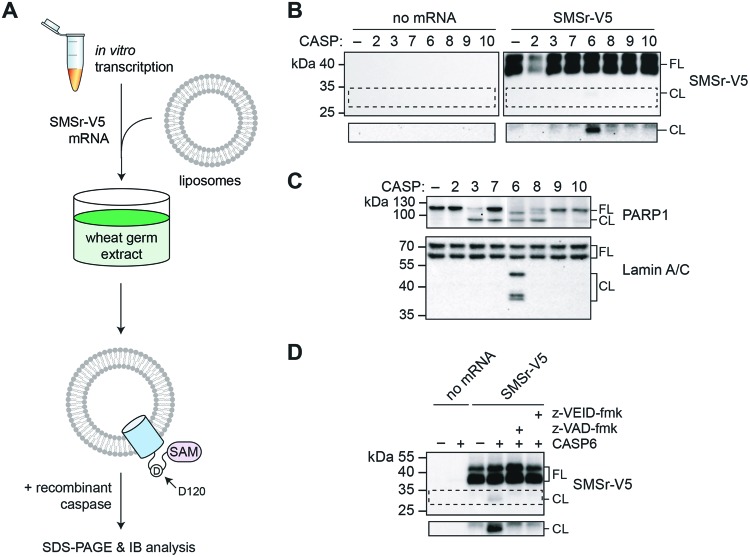
SMSr is a substrate of caspase-6 *in vitro* (**A**) Schematic outline of the wheat germ-based assay for cell-free translation of SMSr-V5 mRNA. Translation reactions were supplemented with liposomes to generate SMSr-V5-containing proteoliposomes. (**B**) Cell-free translation reactions with SMSr-V5 mRNA or no mRNA were incubated with one unit of active recombinant human caspase-2, -3, -6, -7, -8, -9, or -10 for 2 h at 37°C and then immunoblotted using an anti-V5 antibody. Longer exposures of the highlighted areas are shown in the lower panel. (**C**) Lysates of HeLa cells were incubated with one unit of active recombinant human caspases for 2 h at 37°C and immunoblotted using anti-PARP1 and anti-Lamin A/C antibodies. FL, full length; CL, cleaved. (**D**) Cell-free translation reactions with no mRNA or SMSr-V5 mRNA were incubated with two units of active recombinant human caspase-6 for 3 h at 37°C in the presence of 1 mM pan-caspase inhibitor z-VAD-fmk, 100 μM caspase-6 inhibitor z-VEID-fmk or carrier control (DMSO). Reactions were processed as in (D).

Having established that SMSr is a caspase substrate, we next set out to determine which caspase(s) is responsible for SMSr cleavage. As caspases are part of proteolytic cascades characterized by countless cleavage events, identification of specific pairs of caspases and substrates is a challenging task. To circumvent this complexity, we used a previously established wheat germ-based system for the cell-free production of V5-tagged human SMSr [40]. In this system, mRNA encoding SMSr-V5 is translated in wheat germ extract in the presence of unilamellar liposomes (Figure 2A). Next, SMSr-containing proteoliposomes are incubated with active recombinant human caspases and then subjected to immunoblotting using anti-V5 antibody. Importantly, this approach takes advantage of the fact that plants are devoid of any structural and functional homologs of animal caspases [43].

As shown in Figure 2(B), incubation of SMSr-V5 proteoliposomes with recombinant caspase-6 yielded a V5-tagged cleavage product of ∼33 kDa. No cleavage product was observed when SMSr-V5 proteoliposomes were incubated with recombinant caspase-2, -3, -7, -8, -9, or -10, or when incubations were performed with proteoliposomes produced in the absence of SMSr-V5 mRNA. Proteolytic activity of recombinant caspase-3, -6, -7, and -8 was verified by testing their ability to induce cleavage of the caspase substrate PARP1 in HeLa cell lysates. Moreover, immunoblot analysis of caspase-treated HeLa cell lysates indicated that Lamin A/C is a specific substrate of caspase-6 (Figure 2C), in line with previous reports [44-46]. To confirm that SMSr is a substrate of caspase-6, SMSr-V5 proteoliposomes were treated with caspase-6 in the presence or absence of various caspase inhibitors. Pan-caspase inhibitor zVAD-fmk and the caspase-6-specific inhibitor z-VEID-fmk in each case completely blocked caspase-6-mediated cleavage of SMSr (Figure 2D). Together, these results indicate that SMSr is a specific substrate of caspase-6 *in vitro*.

### SMSr is a substrate of caspase-6 *in cellulo*

**Figure 3:**
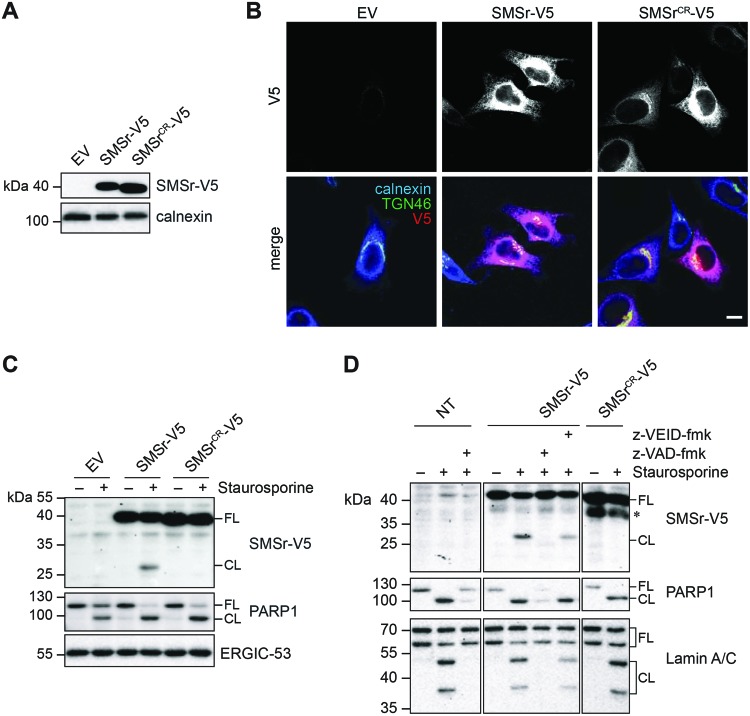
Staurosporine-induced cleavage of SMSr is sensitive to caspase-6 inhibitors (**A**) Total lysates of HeLa cells stably transduced with EV, SMSr-V5, or SMSr^CR^-V5 were subjected to immunoblot analysis using anti-V5 and anti-calnexin antibodies. (**B**) Confocal sections of HeLa cells stably transduced with EV, SMSr-V5, or SMSr^CR^-V5 and immunostained using anti-V5 (red), anti-Golgi marker TGN46 (green), and anti-calnexin (blue) antibodies; bar, 10 μm. (**C**) HeLa cells stably transduced with EV, SMSr-V5, and SMSr^CR^-V5 were treated with 1 μg/ml staurosporine for 6 h, lysed and subjected to immunoblot analysis using anti-V5, anti-PARP1, and anti-ERGIC-53 antibodies; FL, full length; CL, cleaved. (**D**) HeLa control cells or cells stably transduced with SMSr-V5 or SMSr^CR^-V5 were treated with 1.5 μg/ml staurosporine for 6 h in the presence of 20 μM pan-caspase inhibitor z-VAD-fmk, 4 μM caspase-6 inhibitor z-VEID-fmk or carrier control (DMSO). Cells were lysed and subjected to immunoblot analysis using anti-V5, anti-PARP1, and anti-Lamin A/C antibodies.

We next addressed whether SMSr also represents a physiological target of caspase-6 during apoptotic cell death. To this end, we created HeLa cell lines stably expressing V5-tagged versions of SMSr or the caspase-resistant mutant, SMSr^CR^. Expression of SMSr-V5 and SMSr^CR^-V5 was confirmed by immunoblot analysis (Figure 3A). Immunofluorescence microscopy revealed that both enzymes predominantly localized to the ER (Figure 3B), indicating that the mutations that render SMSr caspase-resistant do not interfere with its subcellular distribution. Next, we analyzed the fate of SMSr-V5 and SMSr^CR^-V5 during staurosporine-induced apoptosis. Treatment of cells with staurosporine resulted in proteolytic cleavage of SMSr-V5 but not of its caspase-resistant counterpart, SMSr^CR^-V5 (Figure 3C), hence confirming that Asp118 and Asp120 are the principle caspase cleavage sites in SMSr.

**Figure 4:**
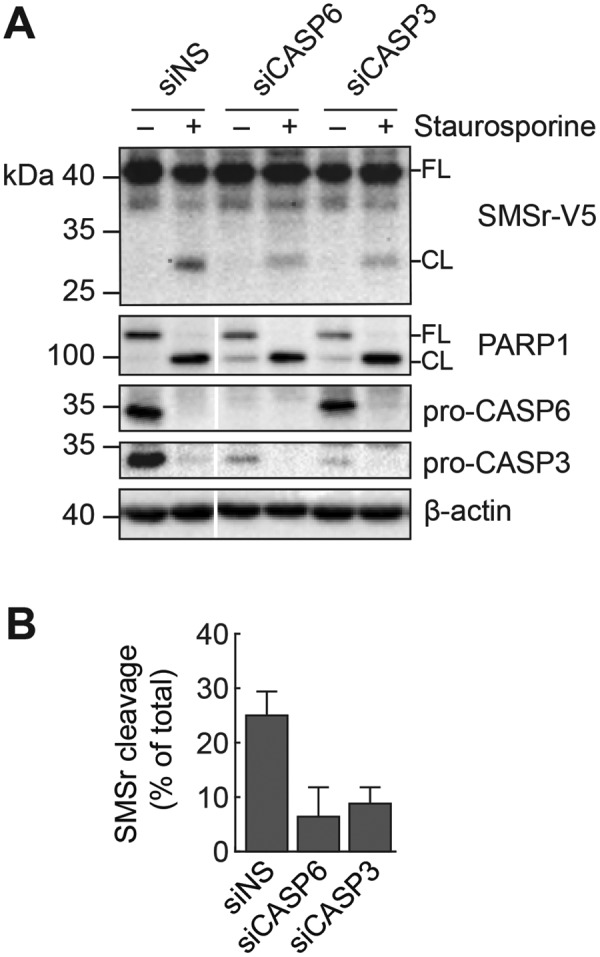
Caspase-6 knockdown reduces SMSr cleavage in staurosporine-treated cells (**A**) HeLa cells stably transduced with SMSr-V5 were treated with non-sense siRNA (siNS) or siRNA targeting caspase-6 (siCASP6) or -3 (siCASP3) for 72 h and then treated with 1.5 μg/ml staurosporine for an additional 6 h. Cells were lysed and subjected to immunoblot analysis using anti-V5, anti-PARP1, anti-caspase-6, anti-caspase-3, and anti-actin antibodies; FL, full length; CL, cleaved. (**B**) Ratio of cleaved SMSr (CL) to total SMSr detected (FL+CL) were calculated from two independent experiments; mean and standard deviation values are denoted.

We then asked whether staurosporine-induced proteolytic cleavage of SMSr in cells is mediated by caspase-6. Addition of pan-caspase inhibitor zVAD-fmk completely abolished cleavage of both SMSr and the caspase-6 substrate Lamin A/C in staurosporine-treated cells, while reducing cleavage of PARP1 (Figure 3D). Addition of caspase-6-specific inhibitor z-VEID-fmk, on the other hand, significantly reduced cleavage of SMSr and Lamin A/C without affecting cleavage of PARP1. Importantly, siRNA-mediated knockdown of caspase-6 also reduced cleavage of SMSr in staurosporine-treated cells (Figure 4A and B). Knockdown of caspase-3 diminished cleavage of SMSr as well; however, this effect is likely indirect given that caspase-3 mediates proteolytic activation of caspase-6 [47-49] and does not recognize SMSr as substrate *in vitro* (Figure 2B). Together, these results indicate that caspase-6 mediates cleavage of SMSr during staurosporine-induced apoptosis.

### Caspase-mediated cleavage of SMSr does not sensitize cells toward apoptotic stimuli

**Figure 5:**
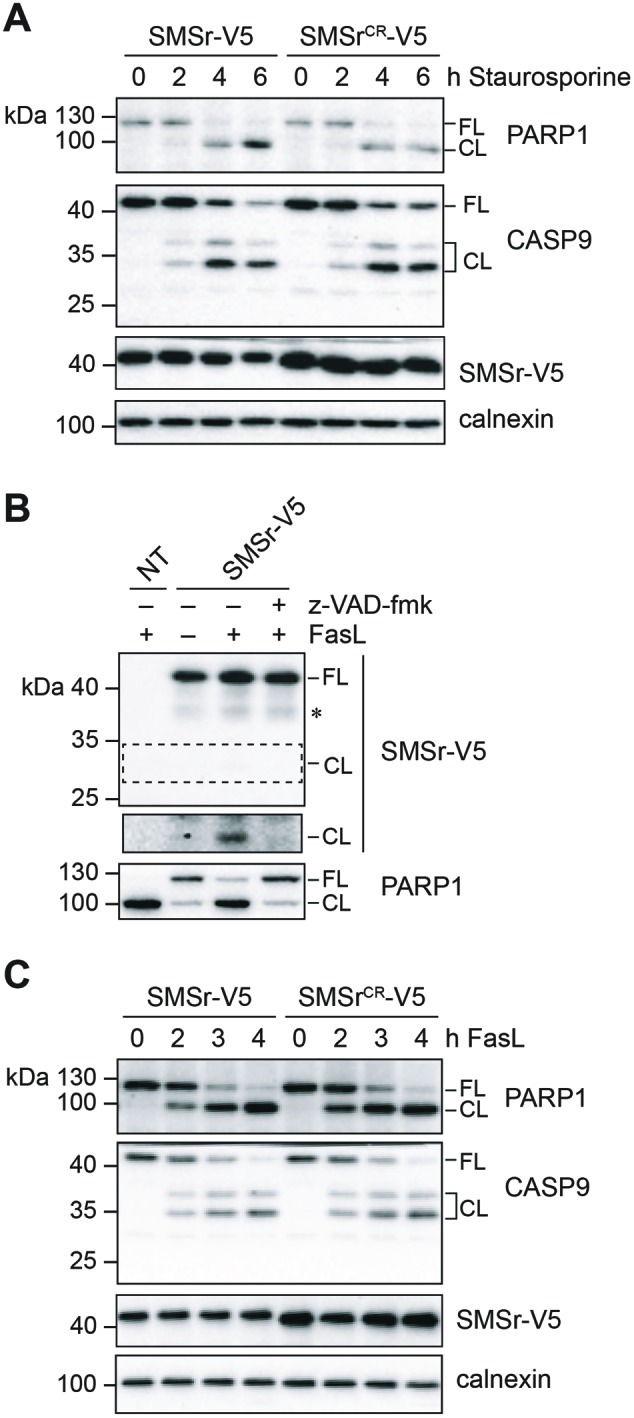
Caspase-mediated cleavage of SMSr does not significantly contribute to staurosporine- or FasL-induced apoptosis (**A**) HeLa cells stably transduced with SMSr-V5 or SMSr^CR^–V5 were treated with 1 μg/ml staurosporine for the indicated time, lysed and subjected to immunoblot analysis using anti-PARP1, anti-caspase-9, anti-V5, and anti-calnexin antibodies; FL, full length; CL, cleaved. (**B**) HeLa cells transiently transfected with SMSr-V5/polyHis were treated with 100 ng/μl FasL and 18.75 μM cyclohexamide for 4 h in the presence or absence of 20 μM z-VAD-fmk. Cells were solubilized with detergent and subjected to Ni^2+^-NTA affinity chromatography. Eluates were immunoblotted using anti-V5 antibodies (top). Total detergent extracts were immunoblotted using anti-PARP1 (bottom); NT, non-transfected. (**C**) HeLa cells stably transduced with SMSr-V5 or SMSr^CR^–V5 were treated with 50 ng/ml FasL and 18.75 μM cyclohexamide for the indicated time, lysed and subjected to immunoblot analysis using anti-PARP1, anti-caspase-9, anti-V5, and anti-calnexin antibodies.

Previous works revealed that cellular ceramide levels rise concomitantly with apoptosis induction in response to staurosporine and various other apoptotic stimuli through activation of sphingomyelinases, stimulation of *de novo* ceramide synthesis, or both [12,20,50]. Interventions that suppress ceramide accumulation render cells resistant to these apoptotic stimuli, suggesting that ceramides play a central role in sensitizing cells to stress-induced apoptosis. Our present data indicate that SMSr loses its N-terminal SAM domain due to cleavage by caspases in staurosporine-treated cells. As SMSr requires its SAM domain to suppress ceramide-induced cell death [39], we wondered whether caspase-mediated cleavage of the enzyme serves to sensitize HeLa cells to staurosporine and other apoptotic stimuli. However, stable expression of the caspase-resistant form of SMSr had no obvious impact on the time-resolved progression of caspase-9 and PARP1 cleavage in staurosporine-treated HeLa cells, indicating that the rate of apoptosis is unaffected by blocking caspase-mediated SMSr cleavage (Figure 5A).

Since SMS1 undergoes caspase-mediated cleavage in leukemia cells treated with the death receptor ligand FasL and because this process has been reported to contribute to FasL-induced apoptosis [35], we next addressed whether SMSr is also cleaved in FasL-treated HeLa cells. As shown in Figure 5(B), treatment with FasL resulted in proteolytic cleavage of SMSr-V5, yielding a V5-tagged fragment of ∼33 kDa. Moreover, cleavage of SMSr was blocked by treating cells with z-VAD-fmk, indicating that this process is mediated by caspases. However, progression of FasL-induced apoptosis was not affected by stable expression of the caspase-resistant form of SMSr (Figure 5C). Collectively, these data suggest that caspase-mediated cleavage of SMSr does not significantly contribute to either staurosporine- or FasL-induced apoptosis in HeLa cells.

## Discussion

CPE synthase SMSr/SAMD8 is an ER-resident suppressor of ceramide-mediated mitochondrial apoptosis that requires its N-terminal SAM domain to exert its anti-apoptotic activity. As several negative regulators of apoptosis are cleaved by caspases during apoptosis induction, we addressed whether SMSr is a target of the apoptotic machinery. In here, we demonstrate that SMSr is a novel and specific substrate of the executioner caspase-6. We provide evidence that in HeLa cells undergoing staurosporine or FasL-induced apoptosis, SMSr is cleaved by caspase-6 at a conserved aspartate located downstream of the SAM domain and upstream of the enzyme’s first membrane span. While this finding is in line with a role of SMSr as negative regulator of ceramide-induced cell death, addressing the physiological relevance of caspase-6-mediated cleavage of SMSr will require further investigations.

Both staurosporine and FasL trigger a caspase-mediated release of the N-terminal SAM domain of human SMSr in HeLa cells. Site-directed mutagenesis revealed that cleavage primarily occurs at Asp120, a residue conserved in SMSr homologs from human to zebrafish. In human SMSr, some residual cleavage also occurs at Asp118. However, this residue is not conserved in the mouse homolog. Taking advantage of human SMSr produced in a wheat germ-based cell-free translation system that lacks endogenous caspase-like activities [43], we were able to reconstitute proteolytic release of the enzyme’s N-terminal SAM domain upon external addition of recombinant caspase-6, but not when adding recombinant caspase-2, -3, -7, -8, -9, or -10. Furthermore, addition of caspase-6-targeting siRNAs or the caspase-6 inhibitor z-VEID-fmk to staurosporine-treated HeLa cells specifically reduced cleavage of SMSr and the caspase-6 substrate Lamin A/C [44]. Together, these findings establish SMSr as a novel target of caspase-6. Whether caspase-6 is the sole caspase responsible for cleaving SMSr during apoptotic cell death remains to be established.

SMSr belongs to the SM synthase family, which also includes the Golgi-resident enzyme SMS1 and the plasma membrane-resident enzyme SMS2 [29,31]. SMS1 has previously been recognized as a target of caspases during FasL-induced apoptosis in Jurkat cells. Contrary to SMSr, SMS1 in FasL-treated cells is cleaved at multiple sites, abolishing the enzyme’s catalytic activity, disrupting *de novo* SM synthesis, and resulting in an accumulation of ceramides [35]. Whereas SMS1 knockdown sensitized cells to FasL-induced apoptosis, SMS1 overexpression had the opposite effect, suggesting that caspase-mediated inhibition of SM production and associated ceramide accumulation are involved in the regulation of FasL-triggered cell death [35]. As SMSr relies on its N-terminal SAM domain to suppress ceramide-induced cell death [39], we considered whether caspase-6-mediated release of this domain may accelerate apoptosis in FasL- or staurosporine-treated cells. However, we did not find any evidence that heterologous expression of a caspase-resistant SMSr mutant influenced progression of apoptotic cell death triggered by these stimuli. While these data suggest that caspase-mediated inactivation of SMSr is functionally unrelated to the regulation of apoptotic cell death, we cannot rule out that overexpression of the enzyme or the coexistence of caspase-resistant and caspase-sensitive SMSr pools in apoptotic cells mask the effect. Therefore, our ongoing efforts are aimed at generating cell-lines in which the proteolytic release of SMSr–SAM can be specifically induced at the level of the endogenous enzyme.

Caspase-6, along with caspase-3 and caspase-7, is classified as an executioner caspase. However, its substrate specificity and activation mechanism are unique among executioner caspases. While caspase-3 and -7 use similar recognition sites to cleave their targets, the recognition sites used by caspase-6 share a high degree of similarity with those of the initiator caspases caspase-8 and -9 [51]. Unlike caspase-3 and -7, caspase-6 can undergo self-activation [ 52,53]. During apoptosis, caspase-6 is translocated to and activated in the nucleus where it cleaves various transcription factors as well as nuclear structural proteins such as lamins, resulting in shrinkage and fragmentation of the nucleus [44,54]. This implies that of all SMSr molecules that populate the ER, only those that reside in the nuclear envelope and expose their SAM-domain containing N-terminal tails in the nuclear matrix would initially be subjected to caspase-6-mediated proteolysis. This may explain our finding that only a relative minor portion of SMSr molecules is cleaved in staurosporine- or FasL-treated cells.

Unlike caspase-3 and -7, caspase-6 activity does not always contribute to apoptotic cell death. For instance, numerous studies point at a critical role of caspase-6 in the development of neurodegenerative diseases [55]. Caspase-6-mediated cleavage of mutant huntingtin protein and amyloid precursor protein (APP) is critical for the onset of Huntington’s disease [56] and Alzheimer’s disease [57] respectively. Moreover, the brains of Alzheimer’s patients have been reported to contain 2–3-fold elevated levels of active caspase-6 [57]. As SMSr constitutes the principle CPE synthase in brain [38], it would be of interest to explore whether its proteolytic cleavage by caspase-6 has any relevance in the pathogenesis of neurodegenerative diseases. In this respect, it is of interest to note our recent observation that the SAM domain of SMSr drives self-assembly of the enzyme into ER-resident trimers and hexamers [58, 58]. Moreover, when expressed on its own, SMSr–SAM readily self-associates into stable polymers [59, 60]. Whether such polymers also form in the brain of patients with elevated levels of active caspase-6 merits further investigation.

## Abbreviations

CPE: ceramide phosphoethanolamine
EV: empty vector
ER: endoplasmic reticulum
FasL: Fas Ligand
SAM: sterile-α motif
SM: sphingomyelin
SMS: sphingomyelin synthase
SMSr: sphingomyelin synthase-related protein
TNF: tumor necrosis factor
